# A New 12q21 Deletion Syndrome: A Case Report and Literature Review

**DOI:** 10.1055/s-0042-1748171

**Published:** 2022-07-21

**Authors:** Alessandra Di Nora, Greta De Costa, Alessia Di Mari, Marco Montemagno, Vito Pavone, Piero Pavone

**Affiliations:** 1 Department of Clinical and Experimental Medicine, University of Catania, Catania, Italy; 2 Department of Radiology, University of Catania, Catania, Italy; 3 Department of General Surgery and Medical Surgical Specialties, Section of Orthopaedics and Traumatology, University Hospital Policlinico-San Marco, University of Catania, Catania, Italy; 4 Department of Clinical and Experimental Medicine, Section of Pediatrics and Child Neuropsychiatry, Hospital “Policlinico G. Rodolico,” Catania, Italy

**Keywords:** 12q21 deletion, pediatry, genetic

## Abstract

Diagnosis in children with physical and intellective anomalies is very challenging because of the wide spectrum of causes. Array-based comparative genomic hybridization (CGH) has acquired an important role in pediatric diagnostic work up. Interstitial deletion of the long arm of chromosome 12 are rare. To date, deletions including the 12q21 region were reported in only 13 patients. The main features are development delay, eyes and central nervous system anomalies, and heart and kidney defects. We describe a 3-year-old boy with a de novo 15 Mb deletion at 12q21.1q21.32, never reported in the last cases. By screening the critical region and reviewing the literature, we identified SYT1, PPP1R12A, and CEP290 such as pathogenetic genes.

## Introduction

Diagnosis in children with physical and cognitive impairment is very challenging because of the wide number of etiological events. Array-based comparative genomic hybridization (CGH) has acquired an important role in diagnostic work up allowing a better definition of the diagnosis. Deletions in the 12q21 region has been rarely reported and so far only 13 cases with this anomaly have been published. We report a 3,1/2years-old boy with development delay, craniofacial dysmorphism, strabismus, muscle mass hypotrophy, pectoral muscle asymmetry, scoliosis, and dysmorphic corpus callosum at the brain MRI. The CGH microarray disclosed a novel 15 MB deletion in the 12q21.1q21.32. Genetic analysis in the parents were normal.

## Case Presentation

**Fig. 1:**
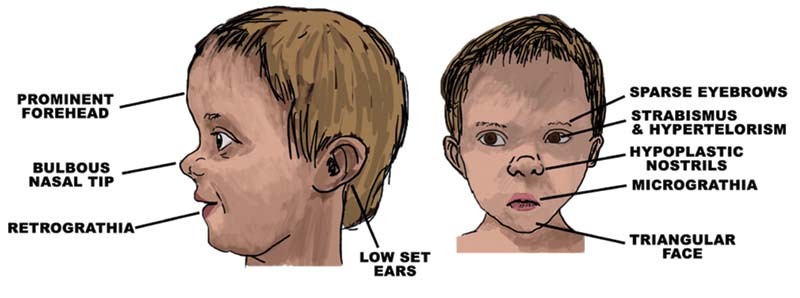
The main clinical features reported in 12q21 deletion children. The imagine was made taking inspiration from our patient and others affected by similar deletion, whose photos are published in the literature. 1 3 8 11 12

**Fig. 2:**
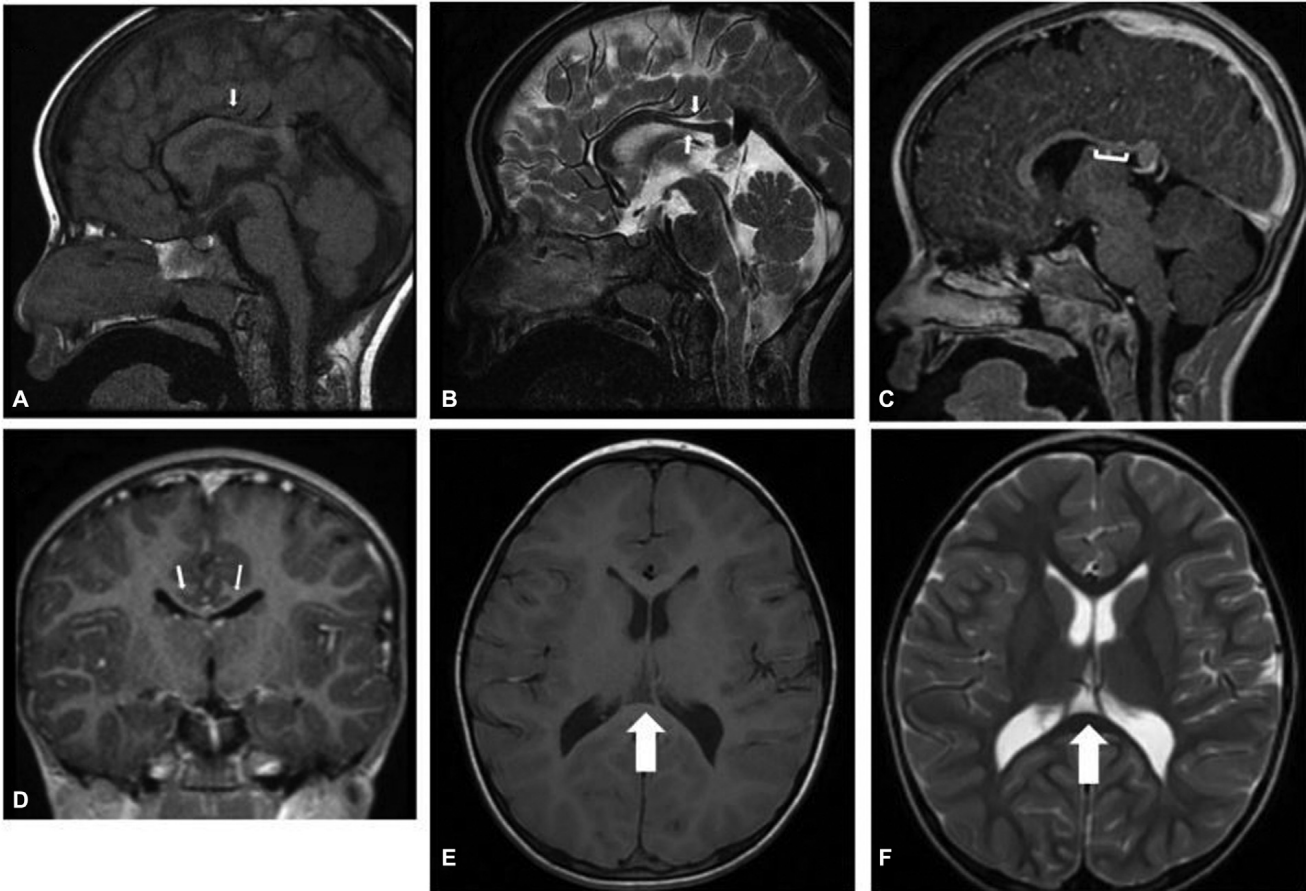
MRI of a 3.5 years-old boy with 12q21 deletion and dysmorphism of the corpus callosum **(A–B–C–D)** . Sagittal T1-weighted MR image **(A)** , Sagittal T2-weighted MR image **(B)** , Sagittal 3D **(C)** and coronal 3D MPRAGE **(D)** images shows dysmorphism of the corpus callosum with appreciable thinning of the middle third and posterior third of the body in relation to the age of the patient (white arrows and white line); Axial T1 **(E)** and T2 **(F)** weighted MR image shows cavum velum interpositum cyst (white arrows).

The proband, a 3.5-year-old boy, is the second child of unrelated parents. The family history is unremarkable. He was born at term by caesarean section for breech presentation, with a weight of 2,700 g. He did not have jaundice or asphyxiation. No teratogenic drug exposures were reported with normal neonatal period. Parents reported a failure to thrive with a regular progression in weight and height, always under 3rd centile. Developmentally, he achieved head support at the age of 5 months, he was able to sit unsupported at the age of 9 months, and walked unsupported at 30 months. His examination reveals prominent forehead, hypertelorism, strabismus, triangular face, low set ears, hypoplastic nostrils, and micro- and retrognathia ( Fig. 1 ). We noted poor muscle weight, asymmetry of the pectoral muscle (left > right), and scoliosis. Control of the sphincters not yet acquired. He is socially responsive, with delayed speech and motor impediment to fine and coarse motor skills. Brain magnetic resonance imaging (MRI) revealed a dysmorphic corpus callosum ( Fig. 2 ). Array-based comparative genomic hybridization (CGH) of DNA extracted from peripheral blood revealed an interstitial deletion of 12q21.1q21.32. The anomaly was 15 Mb. The analysis on his parents was negative.

## Discussion

The first to describe an interstitial deletion of the long arm of chromosome 12 was Meinecke's in the 1987, describing a syndrome with multiple malformations including cleft lip and palate and cardiac abnormalities in 12q13.3q21.1 deletion. 1 Two years later, Watson et al described a 12q15q21.2 deletion in a child with physical abnormalities and development delay. 2 Thirty-two years have passed since these first report;: the reports on this topic increased after the introduction of array-based CGH examination which allowed researchers to extend the phenotype of this disorder. Several molecular mutations have been reported in other patients. 1 2 3 4 5 6 7 8 9 10 11 12 Common features included development delay, clinical dysmorphism, heart defects, and anomalies in the central nervous system. Most of the 12q21 deletion syndrome cases reported in the literature involve the *SYT1* , *PPP1R12A* , and *CEP290* genes.

**Fig 3:**
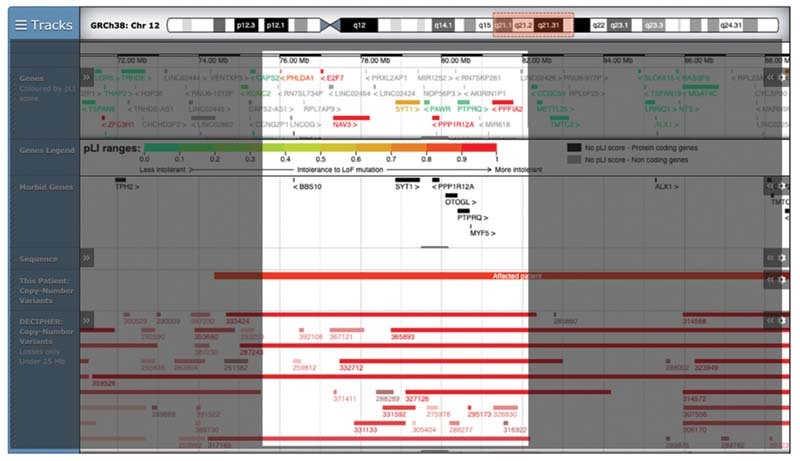
Image modifed from Decipher with the genes involved in the mutation of the proband.

We compared the phenotype with the data available in the publica database DECIPHER ( Fig. 3 ). The main characteristic in common with our child were developmental delay, musculoskeletal abnormalities, and corpus callosum anomalies. A previous study, published in 2020 by Niclass et al described two candidate genes as critical component of the deletion: SYT1 and PPP1R12A. 11 SYT1 encodes an integral membrane protein of postsynaptic vesicles thought to serve as Ca ^2+^ sensors in the process of vesicular trafficking and exocytosis. 13 Mutations in the SYT1 cause neurodevelopment disorder described in a rare syndrome, Baker–Gordon syndrome. They reported 11 individuals affected by infantile hypotonia, congenital ophthalmic abnormalities, childhood-onset hyperkinetic movements disorder, motor stereotypies, and developmental delay. In addition, SYT1 is included as a syndromic gene for the autism spectrum disease in the SFARI database ( Fig. 3 ). Although the patient herein reported carries a very large deletion, the phenotype is consistent with that described in the work by Niclass et al. It underlines that a small region, including the candidate-genes SYT1 and PPP1R12A, can be considered critical and sufficient for the clinical manifestations of 12q21 microdeletion syndrome.

PPP1R12A encodes a regulatory subunit of myosin phosphatase. This enzyme is recently associated in the cellular processes such as cell cycle, gene expression regulation, neurotransmitter release, and even embryonic development. 14

**Fig. 4:**
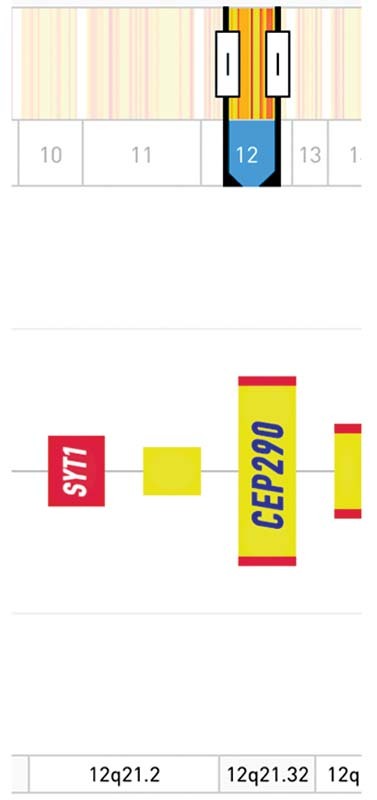
Modified from *SFARI* genes where genes involved with high confidence.

We suppose also CEP290 as one of the main genes for our child. In the literature, there are suggestive evidence in autism reports ( Fig. 4 ). Although the molecular function is playing a role in ciliary transport processes, defects in this gene are associated with several neurologic diseases, for example Joubert's syndrome, Leber's congenital amaurosis, or Meckel's syndrome 15 ( Tables 1 and 2 ).

**Table 1:** Comparing the deletions and phenotypic features of our patient with 15 reported cases with deletion in the region of 12q21

	Deletion type	Dysmorphic features	Development	SNC anomalies	Cardiac	Renal	Musculo skeletal	Other
Study (year)	12q 21.1q21.32		Delayed	CC hypoplasia	Normal	Normal	Scoliosis	
**Meinecke and Meinecke** ( **1987** ) 1	12q13.3q21.1	Present	Delayed	No reported	No reported	No reported	No reported	
Watson et al (1989) 2	12q15q21.2	Present	Delayed	No reported	No reported	No reported	No reported	
Brady et al (1999) 3	12q21.2q23.32	Present	Delayed	No reported	No reported	No reported	Short stature	GH deficit
Rauen et al (2002) 4	12q21.2q22	Present	Delayed	Hydrocephalus	Septal defect	No reported	Normal	
Klein et al (2005) 5	12q21.2q22	Present	Delayed	Mild ventriculomegaly	PDA and PFO	right moderate hydronephrosis and duplication of right collecting system	Scoliosis 2/3 toe syndactyly	Atopic dermatitis, hyperopia, bilateral conductive hearing loss, gastrostomy, bitemporal alopecia, bilateral hydroceles
James et al (2005) 12	12q21.2q22	Present	Delayed	No reported	No reported	No reported	Normal	Skin hyperkeratotic, papular eruption
Schluth et al (2008) 6	12q15-q21.2	Present	Delayed	No reported	Ventricular septal defect	No reported	2/3 toe syndactyl mild pectus excavatum brachydactyly	Gastro esophageal reflux treated by Nissen intervention
Matsumoto et al (2014) 7	12q21.2-q21.33	Present	Delayed	Mild ventriculomegaly and hypoplasia of the CC	No reported	No reported	Mild spastic diplegia	Sleep disturbance
Oliveira et al (2015) 8	12q21.2q22	Present	Delayed	Anomalous subcortical white matter hyperechogenicity ventriculomegaly and hypoplasia of CC	No reported	right vesicoureteral reflux and left renal pelvis dilation	2/3 toe syndactyly 4th/5th clinodactyly	Axial hypotonia hyperkeratosis pilaris and ulerythema ophryogenes
Cano et al (2016) 9	12q21.1q21.33	Present	Delayed	No reported	No reported	No reported	2/3 toe syndactyly	
McKenna et al (2019) 10	12q21.1q21.33	Present	Delayed	Slight ventriculomegaly	PFO	No reported	No reported	Small left-side hydrocele
Niclass et al (2020) 11 P1	12q21.1q21.3	Present	Delayed	Ventriculomegaly dysmorphic CC and developmental abnormality of the frontal vein	No reported	Horseshoe kidneys	muscle weakness	ataxia, dysarthria, dysmetria surgery for pyloric stenosis, gastroesophageal reflux
Niclass et al (2020) 11 P2	12q21.2q21.31	Present	Delayed	No reported	No reported	No reported	pectus excavatum	autism spectrum disorder

Abbreviation: CC, corpus callosum; GH, growth hormone; PDA, patent ductus arteriosus; PFO, patent forame ovale; SNC, central nervous system.

**Table 2:** Clinical features of previous patients and our case

Clinical features	Previous case	Our patient
Hypertelorism	10/13	+
Hypothelorism	2/13	₋
Low set ears	11/13	+
Short neck/webbed neck	3/13	₋
Retrognathia	7/13	+
Micrognathia	9/13	+
Prominent forehead	10/13	+
Bulbous nasal, short nose	8/13	+
